# An improved Wolf pack algorithm for optimization problems: Design and evaluation

**DOI:** 10.1371/journal.pone.0254239

**Published:** 2021-08-26

**Authors:** Xuan Chen, Feng Cheng, Cong Liu, Long Cheng, Yin Mao

**Affiliations:** 1 Design and Art Branch, Zhejiang Industry Polytechnic College, Zhejiang, China; 2 School of Mathematics, Southwest Jiaotong University, Chengdu, China; 3 School of Computer Science, Shandong University of Technology, Zibo, China; 4 School of Control and Computer Engineering, North China Electric Power University, Beijing, China; 5 Department of Computer Science, Fordham University in the New York City, New York, New York, United States of America; Torrens University Australia, AUSTRALIA

## Abstract

Wolf Pack Algorithm (WPA) is a swarm intelligence algorithm that simulates the food searching process of wolves. It is widely used in various engineering optimization problems due to its global convergence and computational robustness. However, the algorithm has some weaknesses such as low convergence speed and easily falling into local optimum. To tackle the problems, we introduce an improved approach called OGL-WPA in this work, based on the employments of *O*pposition-based learning and *G*enetic algorithm with *L*evy’s flight. Specifically, in OGL-WPA, the population of wolves is initialized by opposition-based learning to maintain the diversity of the initial population during global search. Meanwhile, the leader wolf is selected by genetic algorithm to avoid falling into local optimum and the round-up behavior is optimized by Levy’s flight to coordinate the global exploration and local development capabilities. We present the detailed design of our algorithm and compare it with some other nature-inspired metaheuristic algorithms using various classical test functions. The experimental results show that the proposed algorithm has better global and local search capability, especially in the presence of multi-peak and high-dimensional functions.

## 1 Introduction

With the rapid growth of the complexity of data applications and systems, swarm intelligence has received increasing attentions. Specifically, to solve complex optimization problems in an acceptable time, different algorithms such as Genetic Algorithm (GA), Particle Swarm Optimization (PSO), Ant Colony Optimization (ACO) as well as their variations have been proposed in the last few decades. All the approaches can be broadly decomposed into a population initialization stage followed by an iteration computing process, and they have been widely used on handling numerical and real-world optimization problems in various domains (e.g., engineering and computer systems).

The Wolf Pack Algorithm (WPA) is a typical swarm intelligence algorithm based on the living habits of wolves [1, 2]. Generally, WPA includes three main intelligent behaviors (i.e., wolf’s searching behavior, leader wolf’s calling behavior and fierce wolf’s round-up behavior) and two mechanisms (i.e., the leader wolf’s competition mechanism based on *winner is king* and the wolf pack’s updating mechanism based on *survival of the fittest wolves*). Compared to other swarm intelligence, WPA has demonstrated good capability in global convergence and computational robustness [2]. Although the algorithm has been applied in various practical problems, such as for placement and path selection [3], network management [4], UAV trajectory planning [5], data flow control [6], etc., it still has several issues in handling complex problems because of the following issues.

- *Issue 1*: The population in WPA lacks effective initialization. Generally, an advanced initialization can ensure the uniform distribution of solutions in searching space to some extent, and thus it can provide better solutions for the iterative process in a swarm intelligence. However, WPA initializes the spatial position of the individual wolf pack in a random way, which reduces the population diversity of the algorithm, and is not conducive to the generation of the optimal solution of the algorithm.
- *Issue 2*: The searching wolf in WPA adopts a greedy wandering strategy. However, when the maximum wandering number is set to a large value, the searching wolf will be prone to get into a local optimal solution due to the excessive greed. In this case, it will not be able to continue to expand the exploration space and thus the global search capability of the algorithm of will reduced.
- *Issue 3*: The convergence speed of WPA is fast at early stages of computing, but has a significant reduction at the later stages. This is mainly because the effect of fierce wolf’s round-up behavior, which will lead to the local optimization of the algorithm and the poor local search effect and reduce the performance of the algorithm.
- *Issue 4*: In WPA, a wolf is randomly selected to be the leader. If its target function value is smaller than that of other wolves, the position of the leader wolf will be replaced. However, updating the leader wolf in such a way will easily result in the whole algorithm falling into a local optimum. Moreover, the lack of a leader wolf’s influence will also reduce the stability of the algorithm.

In fact, to achieve better optimization performance, various works have been proposed to improve the WPA approach, especially for the first three issues described above. For example, the work [7] has introduced different approaches for WPA to improve its population diversity. For the second issue, the study [8] has pointed out that applying chaos strategy in WPA’s searching behavior can effectively avoid falling into local optimum. Moreover, the paper [9] has presented how to use Levy behavior to optimize the round-up behavior for the third problems. In comparison, to the best of our knowledge, the fourth issue has not been studied yet.

To improve the WPA method in a comprehensive way, in this work, we introduce an optimized approach called OGL-WPA, i.e., an improved *WPA* based on the *O*pposition-based learning and *G*enetic algorithm with *L*evy’s flight. Specifically, we focus on leveraging the existing intelligent techniques (i.e.,opposition-based learning, GA and Levy’s flight) to optimize WPA. We believe that our design and the evaluation conducted in the work as well as the reported results are of value to the community as a basis for understanding the merits of our algorithm. Generally, the contributions of this paper are summarized as follows.

- To improve the performance of WPA, we introduce an optimized approach called OGL-WPA, with the seamless integration of three intelligent techniques: opposition-based learning, Genetic algorithm and Levy’s flight.
- We present the details on how to use the three intelligent techniques to optimize WPA as well as the general implementation flow of OGL-WPA.
- Our evaluation over classical test functions demonstrate that OGL-WPA has better local and global search capability, compared to some popular swarm intelligence algorithms. Specifically, it has significant advantages in solving multi-peak and high-dimensional problems.

The remainder of this paper is organized as follows. The related work of WPA and its principle are introduced in Section 2 and Section 3 respectively. The proposed OGL-WPA is presented in Section 4. The relevant experimental results are reported in Section 5 and the conclusions of this work are summarized in section 6.

## 2 Related work

In this section, we mainly present the related work of WPA. Since another wolf related swarm intelligence called Grey Wolf Optimizer (GWO) [10] becomes increasingly popular in the current research, we also give the relevant studies of GWO.

### 2.1 WPA related works

#### 2.1.1 Optimization of WPA

Xiu *et al.* [9] proposed a tent chaotic map and Levy flight in WPA. Experimental results showed that the optimized algorithm has faster convergence speed and higher precision. Li *et al.* [8] proposed a Chaos-based WPA, which can effectively improve the local search ability and had good development and balance ability. Simulated results showed that the algorithm has higher effectiveness and robustness.

#### 2.1.2 Hybrid algorithms based on WPA

Chen *et al.* [7] proposed a hybrid algorithm based on WPA and differential evolution algorithm (DEA). The DEA was introduced into the individual of WPA to increase the individual diversity of the population and improve the local search ability. Simulation experiments showed that the algorithm performance is better than genetic algorithm (GA), DEA, PSO and artificial bee colony (ABC) algorithms. Based on WPA, Chen *et al.* [11] gave a hybrid improved algorithm WPA-PSO and demonstrated that it can improve the performance effectively.

#### 2.1.3 Applications of WPA

Jiang *et al.* [12] showed that WPA can improve the efficiency of route planning. Dong *et al.* [13] proposed a hybrid optimization algorithm based on wolf pack search and local search (WPS-LS) in business travel problems. They found that it had better robustness and planning effect by simulations. Liang *et al.* [14] proposed a cluster cooperative algorithm based on improved wolf’s behavior, which showed that the model can effectively guarantee the efficiency of solving large-scale complex optimization problems and the operational effectiveness of distributed cluster cooperative attack problems. Moreover, the convergence of the algorithm is proved by Markov asymptotic convergence theory. Zhang *et al.* [15] proposed a hybrid prediction model of WPA based on fuzzy clustering and least squares vector machine, and applied this model to electric bus, which obtained high prediction accuracy and stability. Han *et al.* [16] used the WPA for scheduling in the RHFS problem, and the experimental results show that the Wolf pack algorithm can effectively solve the existing scheduling problems in the RHFS problem. Gao et al. [17] proposed a WPA based on quantum coding to solve the 0–1 knapsack problem, it is showed that the algorithm had better global search ability by simulation. Chen *et al.* [18] proposed the optimized WPA for the route planning of the three-dimensional UAV and the route planning accuracy can be greatly increased by simulation.

### 2.2 GWO related works

#### 2.2.1 Optimization of GWO

Gupta *et al.* [19] proposed an improved algorithm RW-GWO based on random walking. Simulated results showed that the performance of the improved GWO improved greatly. Long *et al.* [20] proposed a GWO based on reverse learning, namely RL-GWO. Simulation results showed that this algorithm is an effective and reliable algorithm for solving function optimization problems.

#### 2.2.2 Hybrid algorithms based on GWO

Arora [21] proposed an algorithm based on the fusion of GWA and Crow algorithm–GWOCSA. Simulated results showed that the algorithm has a good ability to solve complicated problems. Al-Tashi *et al.* [22] proposed another algorithm which combined binary-based particle swarm and GWA, it called BGWOPSO. The algorithm found the best feature subset through K-nearest neighbor classifier. Simulated results showed that the algorithm was better than GWO, PSO, GA and other algorithms. Singh *et al.* [23] proposed an improved grey wolf optimization algorithm, which was used to solve the economic power load scheduling problem and achieved good results. Barraza *et al.* [24] proposed a fusion algorithm based on FWA and GWO. The test of the benchmark function showed that the fusion algorithm had better performance. Gaidhane *et al.* [25] created GWO-ABC by combining ABC with GWO. Firstly, the algorithm applied the chaotic strategy for population initialization in the WPA. Then, enhance its exploration ability by applying the information sharing strategy of the bees in the ABC. Simulated results showed that the performance of the improved gray wolf algorithm has been improved. Zhi *et al.* [26] combined the PSO algorithm with the GWO algorithm, which we called PSO-GWO. In the simulated experiments, they used Tent to initialize population, and the best individual idea of PSO is used to update the individual of the wolf group, which can prevent it from falling into local optimum. The presented simulation results showed that PSO-GWO had a better global optimal solution. In addition, Al-Wajih *et al.* [27] proposed an algorithm called HBGWOHHO, which is based on the combination of GWO algorithm and Harris Hawks Optimization. Moreover, Banaie-Dezfouli *et al.* [28] proposed a method called R-GWO, which is constructed by a representative based grey wolf optimizer.

#### 2.2.3 Applications of GWO

Maharana *et al.* [29] applied the GWA and the JAYA algorithm for job scheduling in the workshop. The simulated results showed that the GWA has better performance. Zapotecas-Martnez *et al.* [30] introduced a multi-objective decomposition GWA, which modeled the multi-objective optimization problem in the form of Pareto’s optimal equilibrium optimal solution set. Simulated results showed that the algorithm has higher quality. In order to achieve lower costs, Kaur *et al.* [31] used the GWA to optimize the offload scheduling of programs on the mobile device side. Simulated experiments showed that the algorithm has better scheduling effect. Al-Moalmi *et al.* [32] proposed the use of the GWA for the optimization of virtual machine layout, which can reduce the number and energy of active hosts. Experiments showed that using this algorithm can effectively reduce energy consumption and use CPU and memory resources better. In order to solve large-scale numerical optimization problems, Long *et al.* [33] proposed an optimized GWO, which we also called ERGWO. The algorithm used a nonlinear adjustment strategy of particle swarms for balancing exploration and development. The simulated results showed that the proposed ERGWO can find high-quality solutions with low computational cost and fast convergence.

From the above related work, we can see that the research on GWO is very active, even more than the WPA. However, as we will demonstrate in this work, an improved WPA can actually perform much better than GWO and also its variants.

## 3 Basic idea of Wolf pack algorithm

WPA [2] is an intelligent algorithm which simulates the wolf pack predation in the natural universe. Generally, the individuals in the wolf pack are divided into three types: the leader wolf, the detection wolf and the fierce wolf. Different kinds of wolves undertake their own responsibility, meanwhile they work cooperatively to catch the prey.

There is only one leader wolf in a pack which acts as a decision maker, leading the pack to catch food as quickly as possible. But the leader wolf in a pack is not fixed, any individual in the pack can become the leader wolf, the leader wolf is determined by competition, that is, only the wisest and fiercest wolf in the group can become the leader wolf. A wolf pack has multiple detection wolves, mainly responsible for the search for the location of the prey within the scope of activities, and then conveyed the prey information to the leader wolf. The location of a wolf’s prey is obtained mainly by the scent of its prey. If the scent is stronger, the closer the wolf is to the prey. There are several fierce wolves in a wolf pack, the wolf is mainly responsible for rounding up the prey. After the detection wolf to find the trail of its prey and report it to the wolf, the wolf called down to round up the prey. When prey is captured, it is not evenly distributed to each individual in the pack. The prey is given priority to the most powerful wolf, and then assigned to the lesser wolf, and finally not even to the weakest wolf.

The distribution rules ensure that the competent wolves can survive, and the small and weak wolves are gradually eliminated, which is conducive to the overall survival and development of the wolves. Therefore, the whole wolf colony algorithm mainly includes two mechanisms, the leader wolf generation mechanism and the wolf pack update mechanism, and three behaviors, namely, search behavior, call behavior and round-up behavior.

The hunting area of the wolf pack is a European space called *N* × *D*, where *N* means the total number of individuals in the wolf pack and *D* means the variable dimension of space. The position state of a wolf in a wolf pack is represented by *X*_*i*_ = (*x*_*i*1_,*x*_*i*2_, …, *x*_*iD*_), where *x*_*id*_ represented the location of wolf *i* in the *d*(*d* = 1, 2…*D*) dimension variable space. Note the concentration (target function value) of the scent of prey perceived by each individual in the wolf pack as *Y*_*i*_, then *Y*_*i*_ = *f*(*X*_*i*_)(*i* = 1, 2,…*N*). The goal of the wolf colony algorithm is to find *x*_*i*_ in a given range of variable values to maximize the value of *f*(*x*_*i*_), that is, the maximum value of the target function, which is a function solving optimization problem.

The two mechanisms and three behaviors of the algorithm are given below, and the detailed analysis of the improved algorithm is prepared.

*1) Leader wolf’s generating mechanism*

The leader wolf changes with the process of rounding up and iteration. During each round up, if the target function value of an individual in a wolf pack is greater than that of the leader wolf, the individual immediately replaces the original position of the leader wolf. During the iteration, the target function value of the optimal wolf after each iteration is compared to the value of the leader wolf in the previous generation, and the position of the leader wolf is updated if better. Throughout the process, we note that the location of the leader wolf is *X*_*lead*_ and its corresponding target function value is *Y*_*lead*_.

*2) Wolf pack’s updating mechanism*

The food distribution rules of the wolf pack cause part of small and weak wolves to be starved to death, so in the wolf colony algorithm, to remove the target function value of the worst *R* wolf individual, and then randomly add *R* wolf individual, where *R* ∈ [*N*/(2 × *β*),*N*/*β*] with *β* for the population update ratio coefficient which is artificially set.

*3) Searching behavior*

In the process of searching for prey, the wolves do not send out the whole pack, but choose the best other than the leader wolf to search for prey first. Set the number of detection wolf random take a whole number of $\left[\frac{N}{\alpha +1},\frac{N}{\alpha }\right]$, where *α* is the proportion factor of detection wolf and is artificially set. When a wolf *i*’s current position of the prey concentration value *Y*_*i*_ > *Y*_*lead*_, then *Y*_*lead*_ = *Y*_*i*_; When *Y*_*i*_ < *Y*_*lead*_, the wolf moves the step length of *Step*_*a*_ in each direction of *h*, calculates the target function value after each step and then returns to its original position. The location of wolf *i* in dimension *d* space after moving in direction *p*(*p* = 1, 2, …,*h*) is:

$$\begin{array}{c} x_{id}^{p}=x_{id}+\sin\left(2\pi \times p/h\right)\times Step_{a}^{d}. \end{array}$$ (1)

At this point, Wolf *i* selects the direction of maximum odor concentration and greater than the current position to go further, update Wolf Status to *x*_*i*_ and repeat the search process until the detection Wolf perceived odor concentration value *Y*_*i*_ > *Y*_*lead*_ or the maximum number of iterations.

*4) Calling behavior*

When the leader wolf calls on the nearby fierce wolf to set the *Step*_*b*_ step to the leader wolf position, the position of the *i* wolf in the *d* dimension variable space at iteration *k* + 1 is:

$$\begin{array}{c} x_{id}^{k+1}=x_{id}^{k}+Step_{b}^{d}\times \left(g_{d}^{k}-x_{id}^{k}\right)/\left|g_{d}^{k}-x_{id}^{k}\right|, \end{array}$$ (2)

where $g_{d}^{k}$ is the position of the first wolf of the generation *K* in the *d* dimension space. When attacking prey, if wolf *i* smell the prey odor concentration value *Y*_*i*_ > *Y*_*lead*_, then *Y*_*lead*_ = *Y*_*i*_, the wolf immediately transformed into a leader and launched a call; if *Y*_*lead*_ < *Y*_*i*_, wolf *i* continues to attack until the distance from the leader is less than

$$\begin{array}{c} d_{near}=\frac{1}{D\times \omega }\times \sum_{d=1}^{D}\left|{\text{max}}_{d}-{\text{min}}_{d}\right|, \end{array}$$ (3)

where, *ω* is the distance determinant, and the value range of the *D*-variable is [min_*d*_, max_*d*_].

*5) Rounding-up behavior*

When the wolf is close to the prey, the wolf will jointly explore the wolf to round up the prey, rounding up the behavior as follow:

$$\begin{array}{c} x_{id}^{k+1}=x_{id}^{k}+\lambda \times Step_{c}^{d}\times \left|G_{d}^{k}-x_{id}^{k}\right|, \end{array}$$ (4)

where, λ represents a random number that is uniformly distributed in [−1, 1], $Step_{c}^{d}$ is the attack step size, and the $G_{d}^{k}$ represents the position of the prey in *d* dimension space in iteration *K*, actually the position of the leader wolf as its location. In addition, the relationship within the three steps in the algorithm are as follows: searching step size *Step*_*a*_, attacking step size *Step*_*b*_ and attacking step size *Step*_*c*_).

$$\begin{array}{c} Step_{a}^{d}=Step_{b}^{d}/2=2Step_{c}^{d}=\left|{\text{max}}_{d}-{\text{min}}_{d}\right|/S, \end{array}$$ (5)

where, *S* is the step length factor, which indicates the degree of granularity of searching the optimal solution in the solution space.

## 4 The improved Wolf pack algorithm

Like many other swarm intelligence algorithms, WPA has good global searchinng capability, but it is easy to fall into local optimization and has slow convergence as we have described. To improve the problems, we propose three improvements in our design. Firstly, using opposition-based learning to initialize the population, which can keep the diversity of the population and avoid the algorithm getting into *precocity*. Secondly, the genetic algorithm is used to improve the selection of the leader wolf. Thirdly, the Levy flight mechanism is used to optimize the round-up behavior.

### 4.1 Opposition-based learning for population initialization

The initial population construction strategy refers to the distribution of the initial population in the solution space. If the initial solution is close to the optimal solution, the algorithm can converge faster, so the initial population construction will directly affect the performance of the algorithm. The initial solution of WPA is randomly generated and the initialization method is simple and easy to implement, but it will reduce the efficiency of the algorithm because it does not estimate the individual. Fortunately, we can solve this problem by opposition-based learning strategies. Opposition-based learning [34] is an optimization method used in machine learning. In each iteration of the algorithm, all the reverse solutions of the current solution are given, and the solution is selected in the current solution and the reverse solution facilitates the evolution, reducing the blindness of the algorithm. In this paper, the population initialization strategy based on opposition-based learning is adopted, by searching the current solution and the reverse solution simultaneously, and selecting the better solution as the initial solution, thus probability of finding the best initial solution is increased. The steps are as follows:

- *Step 1*: Create the initial population randomly. The initial population *NP*_1_ = {*x*_1_(*t*), *x*_2_(*t*), …, *x*_*N*_(*t*)} of the random generation algorithm is calculated according to the formula (6),
  $$\begin{array}{c} x_{i}^{j}=x_{\text{min}}^{j}+rand\left(0,1\right)\left(x_{\text{max}}^{j}-x_{\text{min}}^{j}\right). \end{array}$$ (6)
  where $x_{i}^{j}$ represents the *i*th individual in dimension *j*, *i* is in the range [1, *N*], *j* is in the range [1, *D*], $x_{\text{min}}^{j}$ and $x_{\text{max}}^{j}$ represent the upper and lower bounds of the space, respectively.
- *Step 2*: Solve the inverse solution. The inverse population *NP*_1_ corresponding to $NP_{op}=\left\{{\tilde{x}}_{1}\left(t\right),{\tilde{x}}_{2}\left(t\right),\ldots ,{\tilde{x}}_{N}\left(t\right)\right\}$ is obtained, and the inverse solution of each individual is given as follows,
  $$\begin{array}{c} {\tilde{x}}_{i}\left(t\right)=x_{\text{min}}^{j}+x_{\text{max}}^{j}-x_{i}^{j}. \end{array}$$ (7)
- *Step 3*: Select the optimal individual. First select the individual *x*_*best*_ with the best value of the target function from *NP*_1_ ∪ *NP*_*op*_, and then calculate the average value $x_{mean}=\left(x_{1}+x_{2}+\ldots +x_{N}+{\tilde{x}}_{1}+{\tilde{x}}_{2}+\ldots +{\tilde{x}}_{N}\right)/2N$ of the solution in *NP*_1_ ∪ *NP*_*op*_, finally compare the corresponding target function values of *x*_*best*_ and *x*_*mean*_ respectively. The individual with the largest value of the target function is the best individual of the population, and record it as *x*_*opbest*_, which is obtained by the following formula,
  $$\begin{array}{c} x_{opbest}=\{\begin{array}{cc} x_{best}, & \text{if}\;f\left(x_{best}\right)>f\left(x_{mean}\right)\\ x_{mean}, & \text{otherwise} \end{array}. \end{array}$$ (8)
  The opposition-based learning algorithm can search in a larger search space and guide individuals to evolve toward the optimal value, thus improve the overall convergence speed of the algorithm.

### 4.2 Genetic algorithm for leader Wolf selection

The goal of WPA is to determine the location of the leader wolf, which is the parameter value of the optimal solution of the algorithm. In WPA, the location of the leader wolf is only determined by the size of the scent concentration of prey, which affects the search ability of the global optimal solution. In this paper, the selection, crossover and mutation of genetic algorithm (GA) are used to select the leader wolf, which makes the new leader wolf have stronger robustness and global optimization, and can accelerate the convergence speed of the algorithm. Set the leader wolf in iteration *g* to $x_{lead}^{g}$, the steps are as follows.

#### 4.2.1 Select actions

According to the GA, the fitness function *γ* is defined as the reciprocal of the objective function. The target function value *γ* corresponding to the individual $x_{i}^{g}$ in the wolves in iteration *g* is defined as follows:

$$\begin{array}{c} \gamma \left(x_{i}^{g}\right)=\frac{1}{f(x_{i}^{g})},\;\;\;\;\;\;i=1,2,\ldots ,N, \end{array}$$ (9)

where formula (9)indicates the value of the fitness function corresponding to the individual in the wolf pack. The probability of a wolf individual being selected is set according to the roulette method, as shown in the formula (10):

$$\begin{array}{c} p_{i}^{g}=\frac{\gamma (x_{i}^{g})}{\sum_{i=1}^{N}\gamma \left(x_{i}^{g}\right)},\;\;\;\;\;\;i=1,2,\ldots ,N, \end{array}$$ (10)

#### 4.2.2 Cross action

Two individuals, $x_{r1}^{g}$ and $x_{r2}^{g}$, are randomly selected by probability $p_{r1}^{g}$ and $p_{r2}^{g}$ for cross operation, as shown in formula (11).

$$\begin{array}{c} \left\{ \begin{array}{c} {\bar{x}}_{r1}^{g}=rx_{r1}^{g}+\left(1-r\right)x_{r2}^{g}\\ {\bar{x}}_{r2}^{g}=\left(1-r\right)x_{r1}^{g}+r)x_{r2}^{g} \end{array}.\right. \end{array}$$ (11)

The value of cross probability *r* is 0.95, and ${\bar{x}}_{r1}^{g}$ and ${\bar{x}}_{r2}^{g}$ are the values after the two individuals cross.

#### 4.2.3 Mutation operation

The leader wolf individual and the two random individuals are performed a mutation operation after cross operation. The variation operation formula (12) is as follows:

$$\begin{array}{c} V_{i}^{g+1}=\{\begin{array}{cc} x_{lead}^{g}+\lambda \times \left|x_{r1}^{g}-x_{r2}^{g}\right|, & rand()<pm\\ x_{lead}^{g}, & otherwise \end{array}, \end{array}$$ (12)

where $V_{i}^{g+1}$ represents the individual after $x_{lead}^{g}$ mutation, the mutation probability *pm* is set to 0.01, *rand*() is a random number between (0, 1), λ is a random factor between (0, 1), when *rand*() < *pm*, $V_{i}^{g+1}$ is the new individual after $x_{lead}^{g}$ mutation. Otherwise it is still $x_{lead}^{g}$.

The target function values of individual $V_{i}^{g+1}$ are compared with $x_{lead}^{g}$ by selecting, crossing, and mutating. If $f\left(V_{i}^{g+1}\right)>f\left(x_{lead}^{g}\right)$, the new leader wolf $V_{i}^{g+1}$ is used. Otherwise $x_{lead}^{g}$ continues to be used.

### 4.3 Levy’s flight for rounding-up behavior

A large number of studies have shown that in the process of hunting in large environment, there are basically *Levy’s flight* search characteristics, that is, long short distance search trajectory and occasional long distance search trajectory interlace. Edwards *et al.* [35] studied the activity characteristics of a particular animal and concluded that in a larger space and a limited search, Levy’s flight is the best search strategy in the local area because it can not only satisfy the search in a small range ensure to obtain the target but also satisfy the coarse search in a large range to avoid local optimum. In order to avoid the local optimization of the algorithm, the Levy’s flight mechanism is introduced in the round-up behavior. The characteristics of Levy’s flight are a random transformation process, and there is no fixed function representation. The study shows that the distribution density function of the variation of the Levy’s flight step can be approximated as follows:

$$\begin{array}{c} Levy\left(s\right)∼{\left|s\right|}^{-1-\beta },0<\beta \leq 2, \end{array}$$ (13)

where *s* is the random step length of Levy’s flight behavior, and according to the literature [36], *s* is expressed as follows:

$$\begin{array}{c} s=\mu /{\left|v\right|}^{1/\beta }. \end{array}$$ (14)

Parameter *μ*, *v* obeys normal distribution, i.e.,

$$\begin{array}{c} \mu ∼N\left(0,\sigma_{\mu }^{2}\right),v∼N\left(0,\sigma_{v}^{2}\right), \end{array}$$ (15)

with

$$\begin{array}{c} \sigma_{\mu }={\left\{\frac{\Gamma (1+\beta )\sin(\pi \beta /2)}{\Gamma \left[\left(1+\beta \right)/2\right]2^{\beta -1}/2}\right\}}^{1/\beta },\sigma_{v}=1. \end{array}$$ (16)

In this paper, the motion steps generated by the Levy’s flight step length are applied to the round-up behavior of the basic wolf algorithm and improved

$$\begin{array}{c} x_{id}^{k+1}=x_{id}^{k}+\lambda \times s\times \left|G_{id}^{k}-x_{id}^{k}\right|, \end{array}$$ (17)

where λ is a random number between [0, 1] and *s* is the moving step length, and the Levy’s flight method can make the wolf algorithm jump out of the local optimization in the later search iteration, enhance the exploration ability of the algorithm, and accelerate the convergence speed of the algorithm.

### 4.4 Algorithm steps

**Fig 1:**
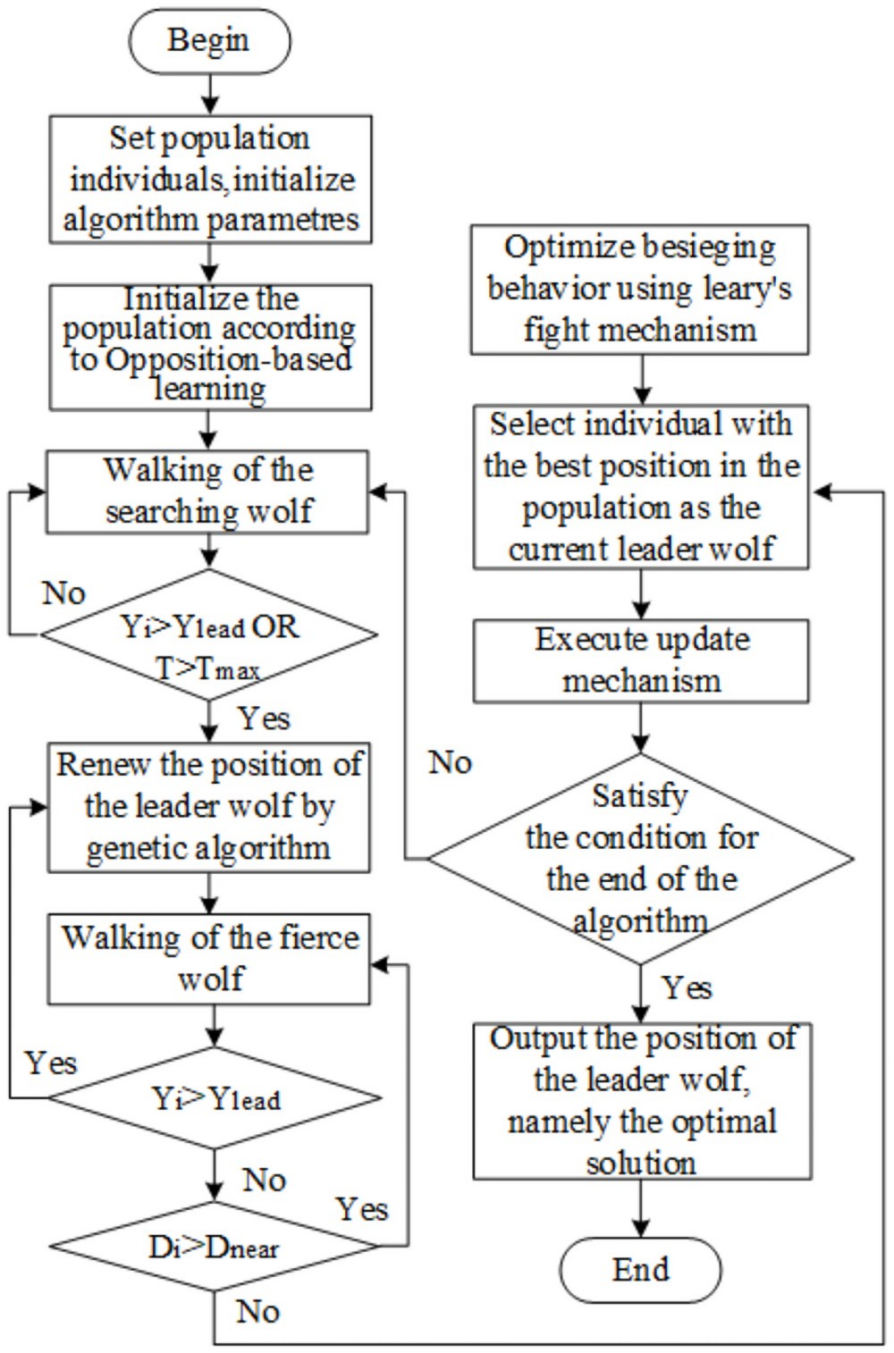
Flow chart of this algorithm.

The implementation workflow of our proposed OGL-WPA is shown in Fig 1. It contains main six steps as following.

*Step 1:* Set the population individual number to be *N*, maximum number of iterations to be *Max*, number of searches for wolves to be *h*, max number of searches for detection wolves to be *T*_max_, search step size, attack step size, and initialize the population according to opposition-based learning.
*Step 2:* Choose the best individual leader wolf according to the description of genetic algorithm. The location is *x*_*lead*_, the target function value is *Y*_*lead*_ and the wolf with the largest target value except the leader wolf is selected as the detection wolf. The formula (1) shall be followed to search the behavior until the *Y*_*i*_ is obtained by a wolf greater than *Y*_*lead*_, or the number of searches reaches *T*_max_.
*Step 3:* Select *M*_*num*_ fierce wolf randomly from the wolf pack except the leader wolf to attack the prey according to the formula (2). When the wolf smell of prey *Y*_*i*_ > *Y*_*lead*_, *Y*_*i*_ = *Y*_*lead*_ instead of the first wolf to initiate the call behavior. Otherwise, the attack will be continued until the distance is less than *d*_*near*_ in formula (3).
*Step 4:* Perform round-ups in accordance with the Levy’s flight mechanism.
*Step 5:* Update the position of the leader wolf according to the leader mechanism and update the population according to the new mechanism.
*Step 6:* Decide whether the optimization is accurate or the number of iterations is reached to *T*_max_. If that case, the location of the output leader wolf is the best solution. Otherwise, return to step 2.

## 5 Experimental simulation

### 5.1 Experiment setting

To illustrate the superiority of our algorithm, we have compared OGL-WPA with some popular swarm intelligence algorithms such as the ant colony optimization (ACO), particle swarm optimization (PSO), Whale Optimization Algorithm(WOA) [37] and LSHADE [38], which are commonly used for solving complex optimization problems, such as task scheduling [39], data communications optimization [40], resource management [41], etc. Moreover, we also have compared OGL-WPA with the GWO algorithm [10, 30] and *β*-GWO algorithm [42], which represent another two latest wolf-related swarm intelligence algorithms. We have implemented all the algorithms with MATLAB 2018b, and run all the tests on a commodity laptop with an I7 CPU running at 1.8Ghz and a 8GB memory. For a general case, the parameters used for each algorithms are presented in Table 1.

**Table 1:** Main parameters of the 7 algorithms.

Algorithms	Main parameters
ACO	Population size is 100;
	pheromone value is 0.005;
	volatility coefficient of pheromone is 0.01;
	path selection probability is 0.5.
PSO	Population size is 100;
	inertia weight is 0.5;
	two learning factors is 0.5;
	random number weight set to be 0.5.
GWO	Population size is 100;
	maximum iterations is 1000;
	alpha = 1000, beta = 2000, GAMMA = 3000.
*β*-GWO	Population size is 100;
	maximum iterations is 1000;
	scaling factor BETA = 0.8, alpha = 1000,
	beta = 2000, gamma = 3000.
WOA	Population size is 100;
	maximum iterations is 1000;
	a decrease linearly from 2 to 0.
LSHADE	Population size is 100;
	maximum iterations is 1000;
	Storage size is 5;
	The optimal selection rate is 0.11.
OGL-WPA	Population size is 100;
	maximum iterations 1000, h is 4, Tmax is 15;
	*Step*_*a*_ is 2, *Step*_*b*_ is 1,
	step factor is 1, λ is 0.5.

### 5.2 Test functions

We have used 12 typical test functions (shown in Table 2) to evaluate the performance of our algorithm. Specifically, these test functions are the basic functions for the design of the CEC competitions [43]. They have both high and low dimensions which can be used to illustrate the comprehensive comparison effects between our algorithm and other six algorithms. The experiment selects the average value, the minimum value, the maximum value and the standard deviation as the evaluation index, in which the maximum value and the minimum value reflect the quality of the solution. The average value reflects the accuracy that the algorithm can be achieved under the given number of iterations, and the standard deviation reflects the convergence speed of the algorithm.

**Table 2:** The used test functions in the evaluation.

No.	Function Name	Test Function	Dimension	Search Space	Optimal Solution
F1	Sphere	$f(x)=\sum_{i=1}^{n}x_{i}^{2}$	2	[-100,100]	0
			5	[-100,100]	0
			10	[-100,100]	0
			30	[-100,100]	0
F2	Schwefel 2.22	$f(x)=\sum_{i=1}^{n}\left|x_{i}\right|+\prod_{i=1}^{n}\left|x_{i}\right|$	2	[-100,100]	0
			5	[-100,100]	0
			10	[-100,100]	0
			30	[-10,10]	0
F3	Schwefel 1.2	$f(x)=\sum_{i=1}^{n}\left(\sum_{j=1}^{i}x_{j}\right)$	2	[-100,100]	0
			5	[-100,100]	0
			10	[-100,100]	0
			30	[-100,100]	0
F4	Schwefel 2.21	$f(x)=\text{max}\left(\text{abs}\left(x_{i}\right)\right)$	2	[-10,10]	0
			5	[-10,10]	0
			10	[-10,10]	0
			30	[-100,100]	0
F5	Rosenbrock	$f(x)=\sum_{i=1}^{n-1}\left[100{\left(x_{i+1}-x_{i}^{2}\right)}^{2}+{\left(x_{i}-1\right)}^{2}\right]$	2	[-30,30]	0
			5	[-30,30]	0
			10	[-30,30]	0
			30	[-30,30]	0
F6	Step	$|f(x)=\sum_{i=1}^{n}{\left(x_{i}+0.5\right)}^{2}$	2	[-100,100]	0
			5	[-100,100]	0
			10	[-100,100]	0
			30	[-100,100]	0
F7	Rastrigin	$f(x)=\sum_{i=1}^{n}\left(x_{i}^{2}-10\text{cos}\left(2\pi x_{i}\right)+10\right)$	2	[-5.12,5.12]	0
			5	[-5.12,5.12]	0
			10	[-5.12,5.12]	0
			30	[-5.12,5.12]	0
F8	Ackley	$\begin{array}{c} f(x)=20+e-\\ 20\text{exp}\left(-\frac{1}{5}\sqrt{\frac{1}{n}\sum_{i=1}^{n}x_{i}^{2}}\right)-\\ \text{exp}\left(\frac{1}{n}\sum_{i=1}^{n}\text{cos}\left(2\pi x_{i}\right)\right) \end{array}$	2	[-32,32]	0
			5	[-32,32]	0
			10	[-32,32]	0
			30	[-32,32]	0
F9	Griewank	$f(x)=\frac{1}{1000}\sum_{i=1}^{n}x_{i}^{2}-\prod_{i-1}^{n}\text{cos}\left(\frac{x_{i}}{\sqrt{i}}\right)+1$	2	[-600,600]	0
			5	[-600,600]	0
			10	[-600,600]	0
			30	[-600,600]	0
F10	Penalgy 1	$\begin{array}{c} f(x)=\frac{\pi }{n}\left\{10\text{sin}\left(\pi y_{1}\right)+\right.\\ \sum_{i=1}^{n-1}{\left(y_{1}-1\right)}^{2}\left[1+10{\text{sin}}^{2}\left(\pi y_{i+1}\right)\right]+\left(y_{n}-1\right)\left.{}^{2}\right\}\\ +\sum_{i=1}^{n}u\left(x_{i},10,100,4\right),\\ y_{i}=1+\frac{x_{i}+1}{4},\\ u\left(x_{i},a,k,m\right)=\\ \left\{\begin{array}{cc} k{\left(x_{i}-a\right)}^{m}, & x_{i}>a\\ 0 & -a<x_{i}<a\\ k{\left(-x_{i}-a\right)}^{m}, & x_{i}<-a \end{array}\right. \end{array}$	2	[-50,50]	0
			5	[-50,50]	0
			10	[-50,50]	0
			30	[-50,50]	0
F11	Bent Cigar	$f(x)=x_{1}^{2}+10^{6}\sum_{i=2}^{n}x_{i}^{2}$	2	[-50,50]	0
			5	[-50,50]	0
			10	[-50,50]	0
			30	[-50,50]	0
F12	Sumsquares-2	$f\left(x\right)=\sum_{i=1}^{n}ix_{i}^{2}$	2	[-50,50]	0
			5	[-50,50]	0
			10	[-50,50]	0
			30	[-50,50]	0

### 5.3 Experimental results

For the 12 test functions, we just report the comparisons of fitness values of the five algorithms in Figs 2–13. Specifically, the detailed comparisons of the four indicators (i.e., minimum value, maximum value, average value and variance) achieved in the dimensions of 2, 5, 10, and 30 as well as the elapsed time have been given online as an S1 Appendix, the link of which has been given in the support information at the end of this work.

**Fig 2:**
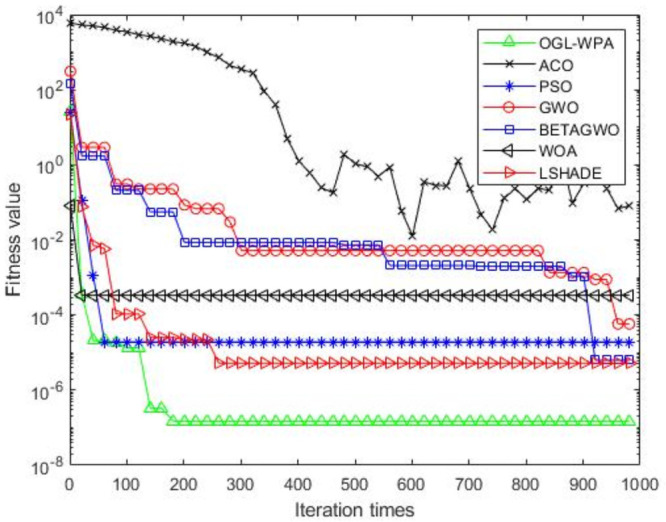
F1 iteration curve.

**Fig 3:**
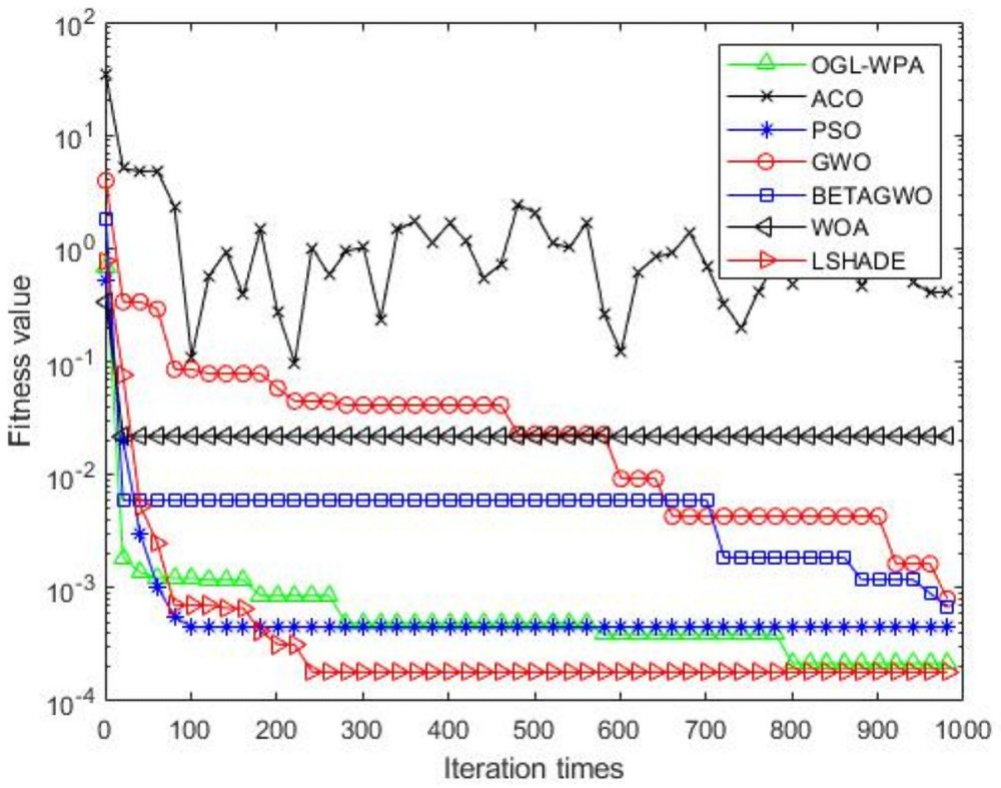
F2 iteration curve.

**Fig 4:**
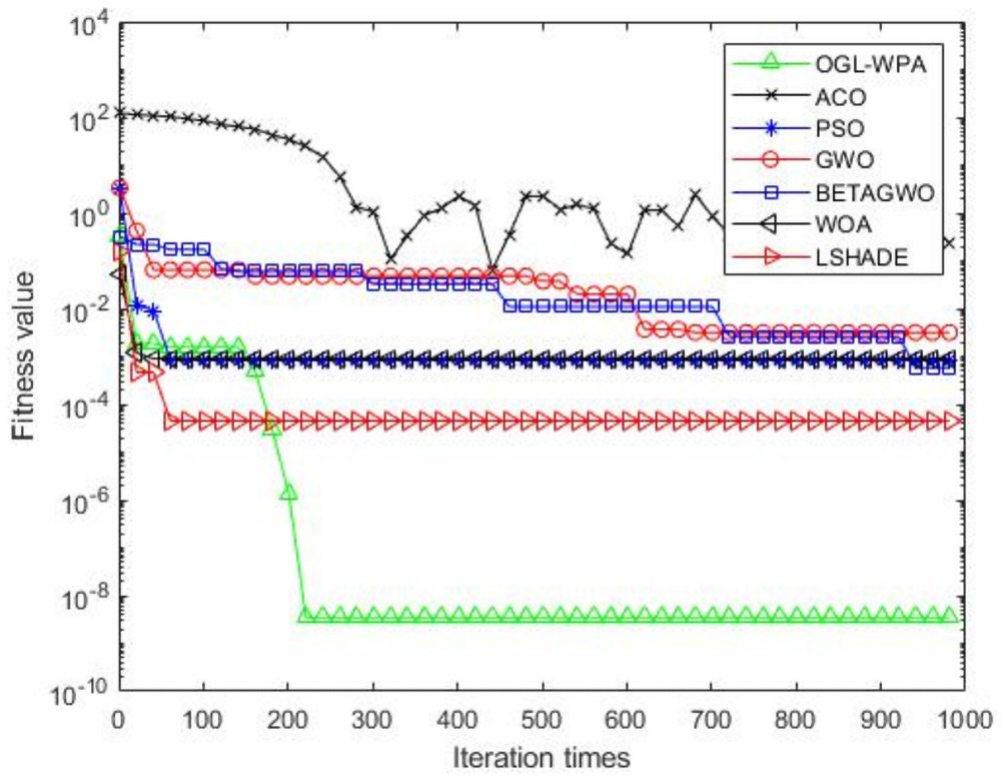
F3 iteration curve.

**Fig 5:**
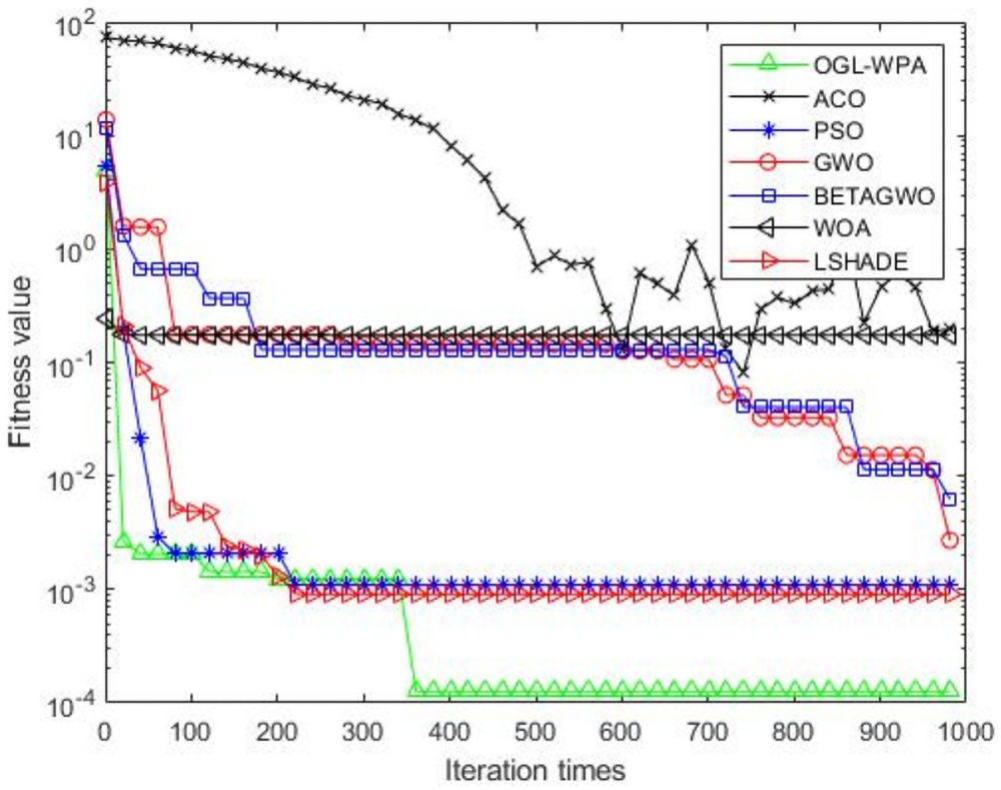
F4 iteration curve.

**Fig 6:**
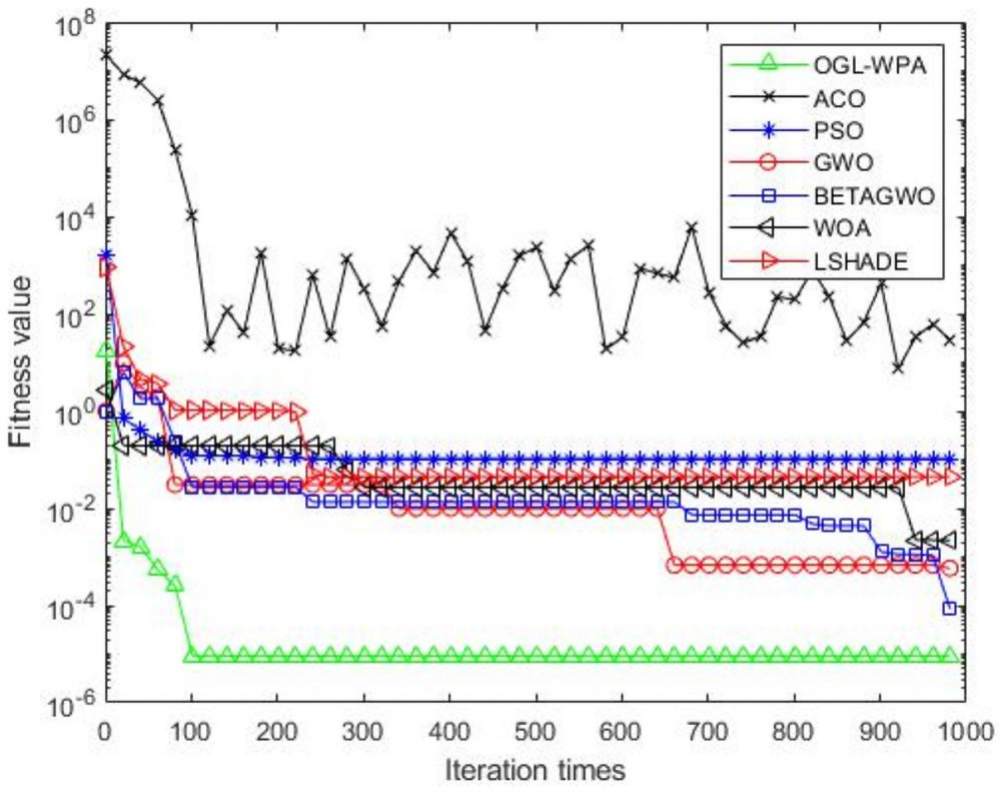
F5 iteration curve.

**Fig 7:**
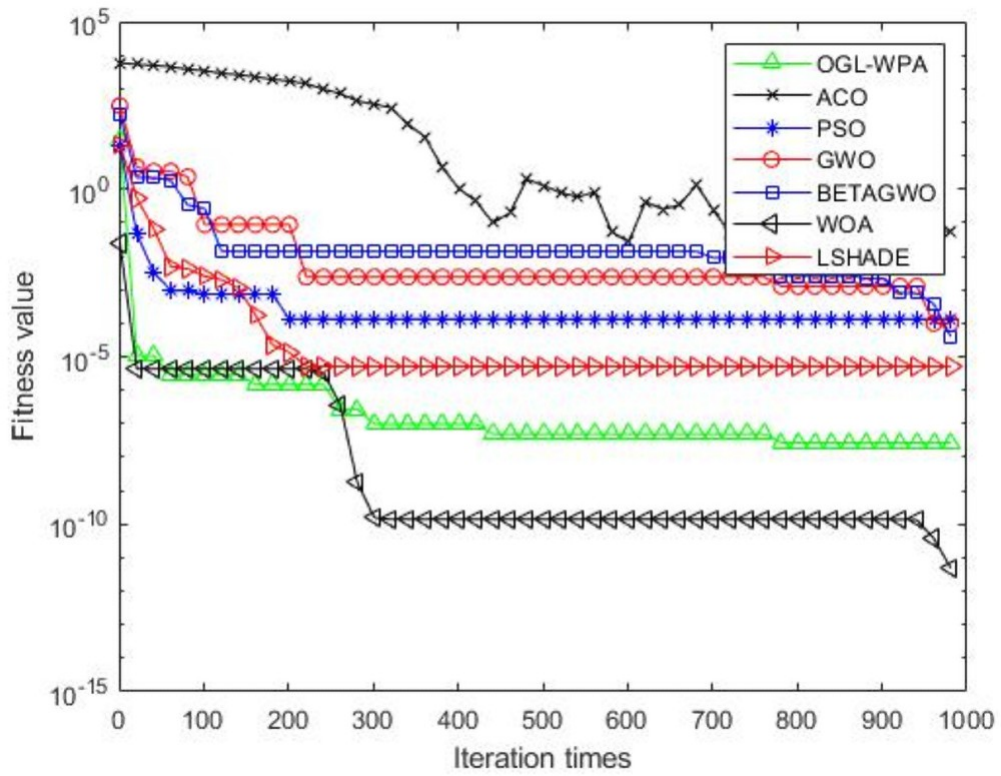
F6 iteration curve.

**Fig 8:**
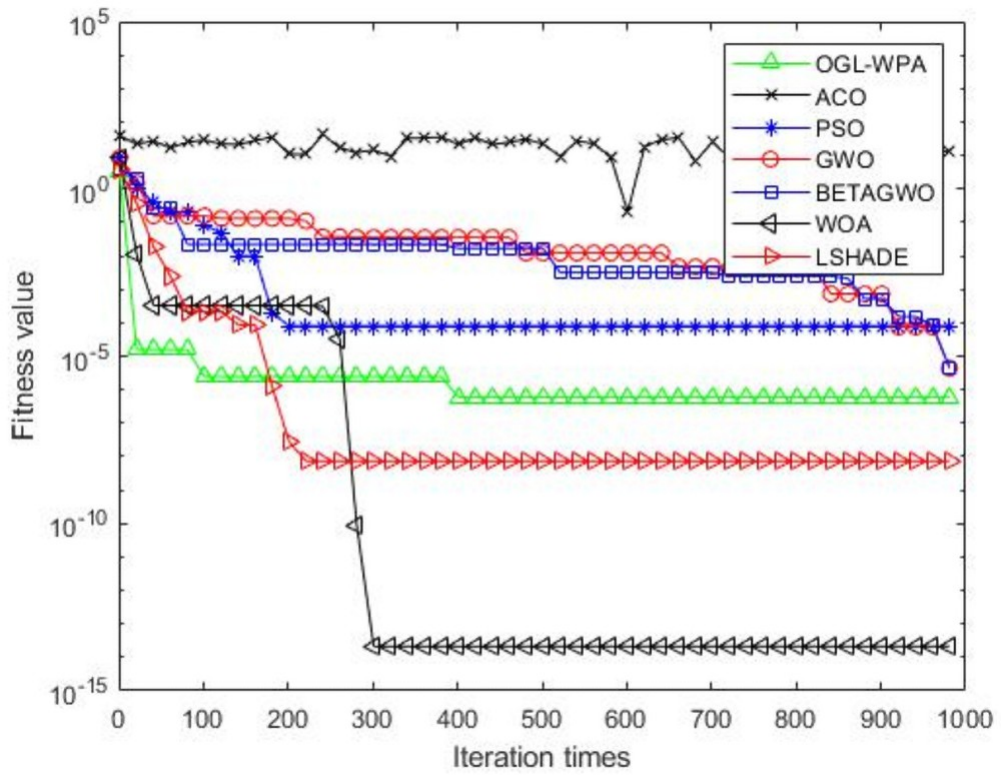
F7 iteration curve.

**Fig 9:**
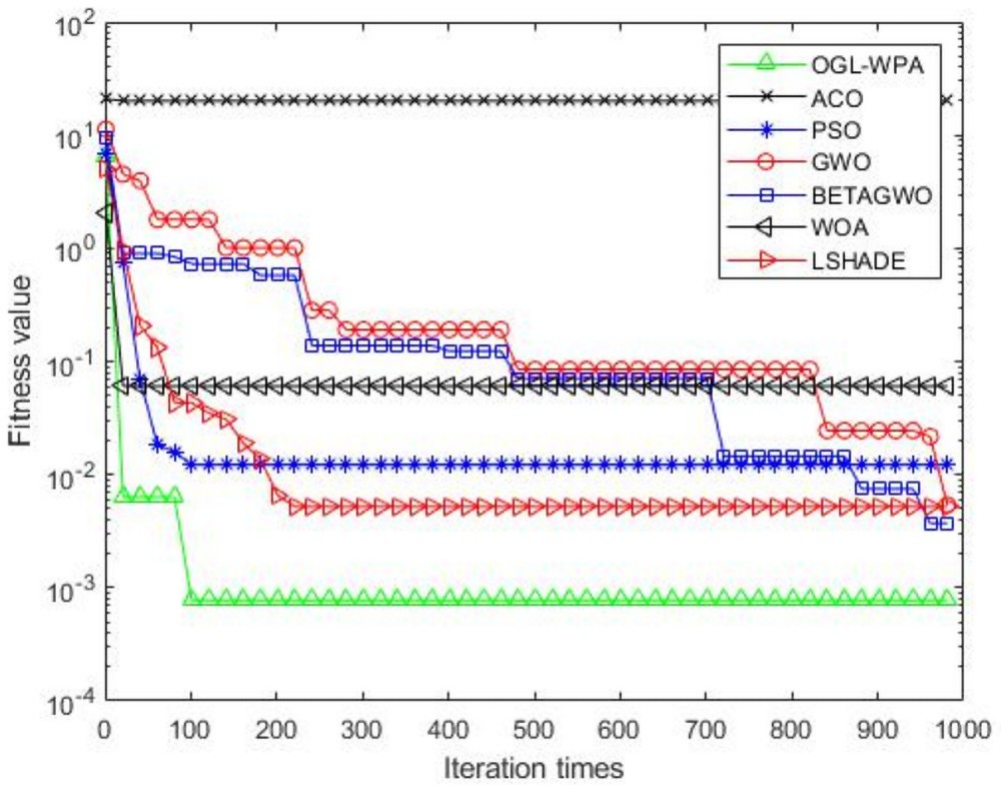
F8 iteration curve.

**Fig 10:**
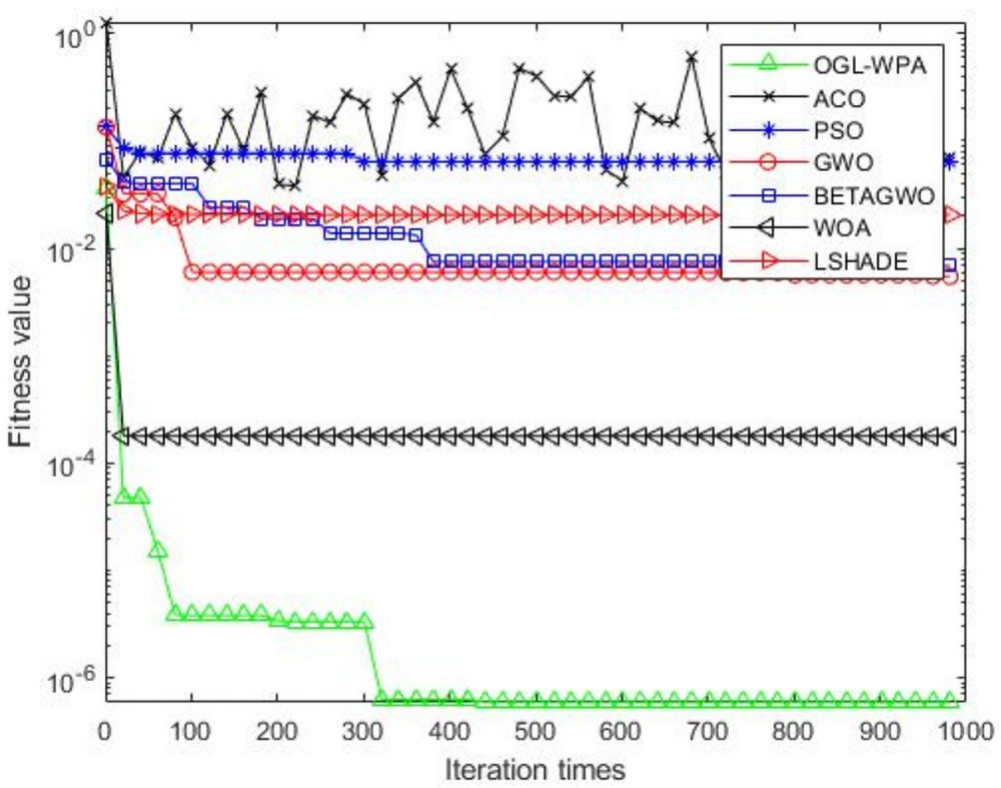
F9 iteration curve.

**Fig 11:**
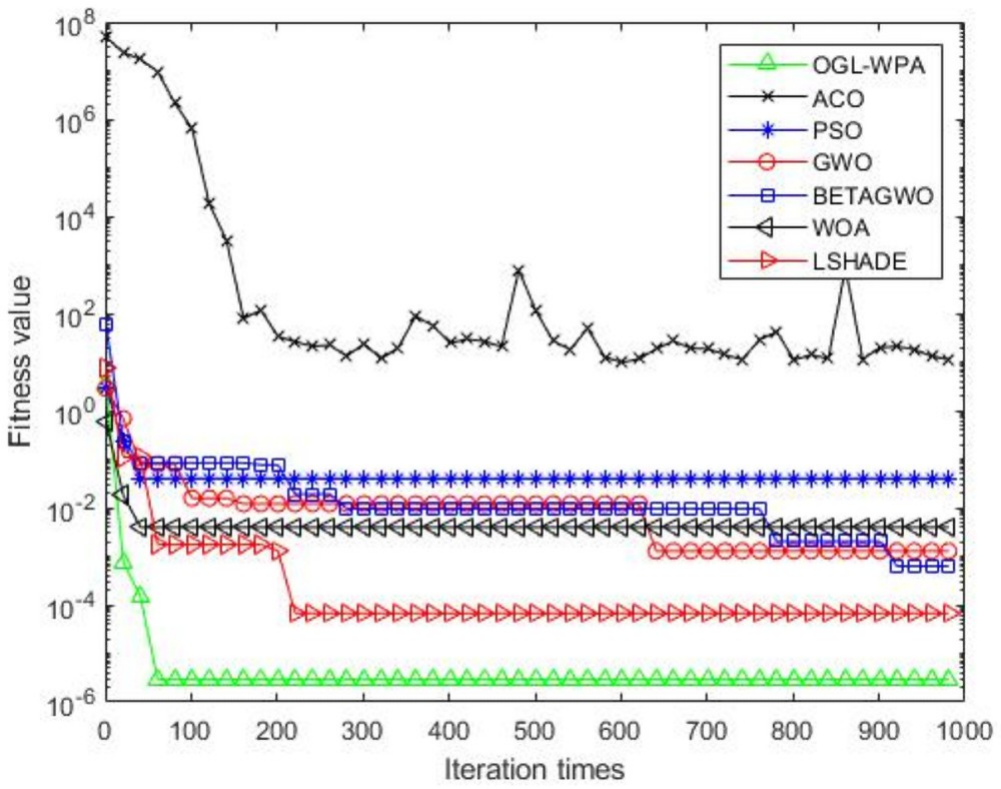
F10 iteration curve.

**Fig 12:**
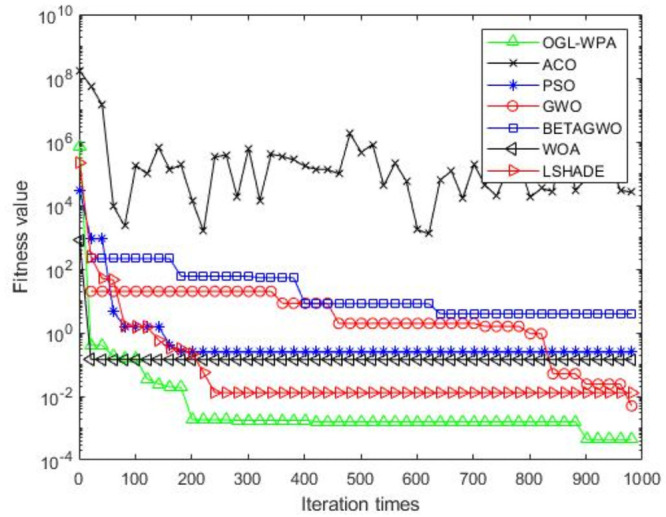
F11 iteration curve.

**Fig 13:**
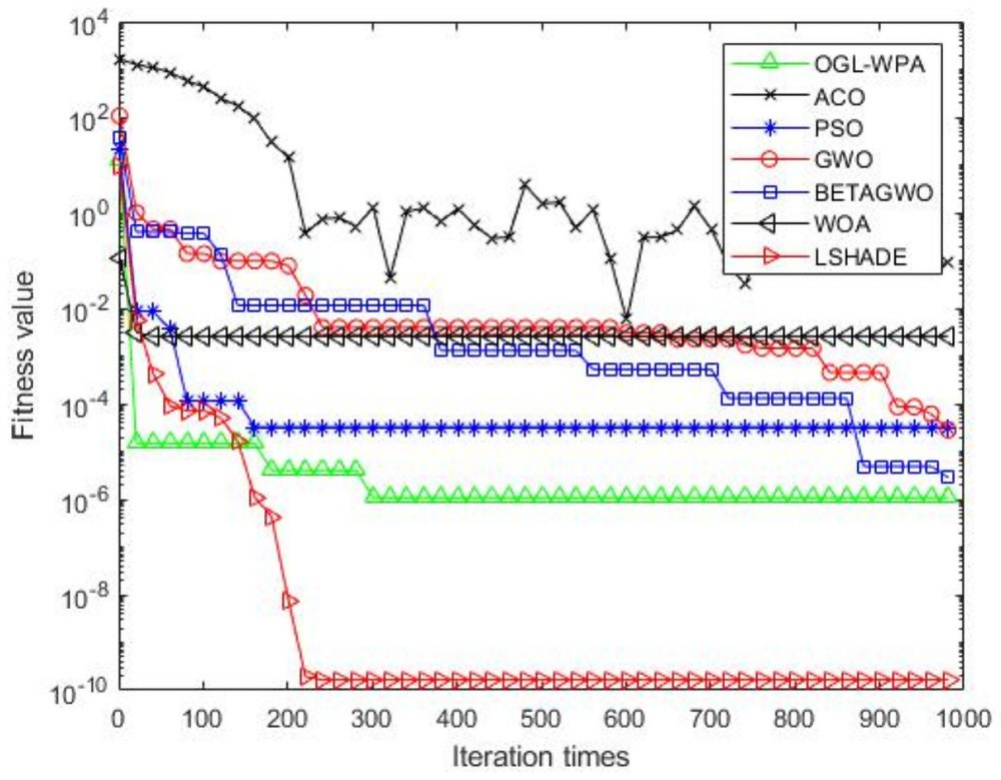
F12 iteration curve.

Fig 2 shows that the convergence of OGL-WPA happens when the number of iteration reaches 180. In comparison, the WOA happens when the number of iteration is 20, LSHADE is 260, PSO happens when the number of iteration is around 150, *β*-GWO is about 950, and the convergence of GWO happens when the number of iteration is around 900. From Fig 2, it can be found that the fitness of ACO decreases at the first half of iterations, and in oscillation process at the latter half, which means it can not converge. Moreover, we can see that our proposed algorithms can achieve the smallest fitness value, compared to other algorithms.

Fig 3 demonstrates that the convergence of OGL-WPA happens when the number of iterations is 800, WOA happens when the number of iteration is 20, LSHADE is 230, the convergence of PSO happens when the number of iteration is around 200. We can also see that fitness values of *β*-GWO and GWO decrease with increasing number of iteration. Although the whole processes of them are both smooth, they can not reach convergence at all. Moreover, for the ACO algorithm, we can see that its fitness value decreases and increases with increasing the number of iterations, demonstrating its poor performance in terms of convergence.

Fig 4 shows that the convergence of OGL-WPA happens when the number of iterations is about 50, WOA is 10, LSHADE is about 80, the convergence of PSO happens when the number of iteration is about 180, the convergence of *β*-GWO happens when the number of iteration is about 900, the convergence of GWO happens when the number of iteration is 760. Similar to previous results, the fitness value of ACO decreases and increases with increasing the number of iterations, which means that it cannot converge in a well way. Compared to the fitness values of all the algorithms, it can be seen that the proposed OGL-WPA can actually achieve the best performance for the optimization problem.

Fig 5 shows that the convergence of OGL-WPA happens when the number of iterations is about 350. Moreover, the convergence of WOA happens when the number of iteration is 10, LSHADE is 210, PSO is about 150. It also shows that the descending trend of *β*-GWO and GWO increases with increasing the numbers of iterations, and finally their fitness values can reach a fix point. Similar to the results above, ACO cannot converge well, and our proposed method can get a smallest fitness value for the test function, compared to all other algorithms.

Fig 6 shows that the convergence of OGL-WPA happens when the number of iterations is about 100. Moreover, WOA convergences when the number of iteration is 10, LSHADE is 220, the convergence of PSO achieves at the beginning of iteration. The results also show the overall descending trend of *β*-GWO and GWO appear with the number of iterations increase, then the whole processes of them are smooth.

Fig 7 shows that the convergence of OGL-WPA happens when the number of iterations is 780. The convergence of WOA is around 300, LSHADE is 230 and the convergence of PSO happens when the number of iteration is around 200. It also shows that the overall descending trend of *β*-GWO and GWO first appears with the increased number of iterations, and then the whole process goes to a smooth status. From Fig 7 it can be also found that the convergence of ACO decreases before the number of iterations is 100, and then the whole process is in oscillation with the increased number of iterations, which means it cannot converge well.

Fig 8 shows that the convergence of OGL-WPA happens when the number of iterations is around 400, WOA is around 20, LSHADE is 230, the convergence of PSO happens when the number of iteration is around 190. It also shows the *β*-GWO and GWO decrease with the number of iterations increase. The whole processes of them are smooth, but they can not reach convergence at all. The ACO alongs with the increase of the number of iterations, but the whole process is in oscillation causing convergence can not be reached.

Fig 9 shows that the convergence of OGL-WPA happens when the number of iterations is around 100, WOA is 10, LSHADE is 220, the convergence of PSO happens when the number of iteration is around 200. It also shows the *β*-GWO and GWO decrease with the increased number of iterations. The whole processes of them are smooth, but they can not reach convergence at all. It can be found that the ACO achieves convergence directly.

Fig 10 shows that the convergence of OGL-WPA happens when the number of iterations is around 450. In comparison, the convergence of WOA happens when the number of iteration is 10, LSHADE is 10, the convergence of PSO happens almost at the beginning. It also shows that the *β*-GWO and GWO decrease with the number of iterations increase. The whole processes of them are smooth, but they cannot reach convergence at all. Moreover, from the results, we can see that the ACO is in the status of oscillation with increasing the number of iterations, which means it cannot converge neither. In contrast, our algorithm can achieve the smallest fitness value in a quick way, demonstrating its strong capability in solving the optimization problems similar to the test function 9.

Fig 11 shows that that the convergence of OGL-WPA happens when the number of iterations is around 50, WOA is 50, LSHADE is 210, and the convergence of PSO happens when the number of iteration is around 200. It also shows the *β*-GWO and GWO decrease with the increased number of iterations, but they cannot reach convergence in a good way. Moreover, the ACO appears the decreasing trend when the number of iterations is about 200, and then it goes into the oscillation status, which demonstrates its poor performance in converge again.

Fig 12 shows that the convergence of OGL-WPA happens when the number of iterations is around 900, WOA is 10, LSHADE is 250, and PSO happens when the number of iteration is 200. Moreover, the convergence of *β*-GWO happens when the number of iteration is about 650. The ACO is in the status of oscillation in the whole process, which means it cannot converge. In addition, we can see that our algorithm can get the best fitness value again, compared to other approaches.

Fig 13 shows that the convergence of OGL-WPA happens when the number of iterations is around 900, WOA is 10, LSHADE is 220, PSO is 180. Moreover, the convergence of *β*-GWO happens when the number of iteration is about 900. Again, the ACO is in the oscillation status with increasing the number of iterations, demonstrating a poor convergence performance. Although the LSHADE can achieve the best performance in terms of fitness values, our method can get the second best result.

Generally, from the results reported in Figs 2–11 above, we can see that PSO is better than OGL-WPA on the convergence speed in functions F1, F2, F4, F5, F6, F7, F9, F10, F11 and F12. However, the fitness value of PSO is obviously lower than OGL-WPA. Moreover, GWO and *β*-GWO have done improvements on WPA, but they always perform worse than OGL-WPA in all the functions. In the meantime, WOA and LSHADE have done improvements on WPA, but they always perform worse than OGL-WPA in all the functions except F2, F6, F7, F12. Specifically, our proposed method can always converge well and can also achieve a good result for all the 12 test functions, demonstrating its advantages in processing different optimization problems.

Tables 3 and 4 report the running time of each algorithm in the presence of the 12 test functions. From the results there, we can see that the computational time of the proposed algorithm in this paper is generally longer than the simple algoritihm, such as ACO, PSO, GWO. However, it can converge well and always get a better fitness value. On the other hand, compared to the advanced algorithsm such as BETAGWO, WOA and LSHADE, the computational overhead of our algorithm is generally samller. In addition, we also have performed statistical analysis of our results and reported the results in Table 5. There, we have used the Wilcoxon test, and the R+ and R- [44] represent the maximum and minimum sum ranks, respectively. The parameters of Wilcoxon test are set to 0.01 and 0.05. From the results presented there, we can see that in all cases, the R+ value provided by OGL-WPA is higher than R-. Therefore, we can say that OGL-WPA can indeed achieve a better performance in generally, compared to other methods.

**Table 3:** Computational time of each algorithm—Part I.

Algo.	Dim.	F1	F2	F3	F4	F5	F6
**OGL-WPA**	2	1.424	2.036	2.39	1.287	1.507	1.204
	5	2.647	4.038	8.249	2.742	6.084	3.137
	10	4.863	7.81	26.695	5.396	13.011	4.96
	30	15.95	27.58	236.85	18.159	75.258	17.927
**ACO**	2	0.289	0.367	0.611	0.284	0.325	0.228
	5	0.646	0.717	1.382	0.629	1.119	0.595
	10	1.239	1.387	3.858	1.27	1.921	1.672
	30	3.361	3.715	22.7	3.994	8.2	3.702
**PSO**	2	0.192	0.172	0.279	0.161	0.21	0.148
	5	0.359	0.346	0.807	0.338	0.527	0.397
	10	0.995	0.99	2.484	0.683	1.176	0.732
	30	2.247	2.711	16.536	1.931	5.198	1.923
**GWO**	2	0.255	0.206	0.302	0.191	0.34	0.223
	5	0.521	0.545	1.005	0.536	0.694	0.531
	10	1.098	1.239	2.908	1.198	1.619	1.174
	30	4.475	4.778	19.29	4.782	7.936	4.793
**BETAGWO**	2	0.734	1.083	1.156	0.783	0.808	0.924
	5	4.153	4.139	5.847	4.004	4.605	4.198
	10	12.582	12.176	14.755	13.709	15.659	14.997
	30	103.31	104.15	126.333	102.097	113.266	145.464
**WOA**	2	1.050	1.518	1.564	0.954	1.308	1.143
	5	5.103	4.826	6.885	5.090	5.877	5.560
	10	16.386	15.248	18.926	17.426	22.179	17.885
	30	134.963	150.652	188.610	147.939	135.533	161.436
**LSHADE**	2	2.304	2.693	3.02	1.975	2.64	2.337
	5	13.057	10.409	17.162	11.368	13.144	14.673
	10	32.532	34.434	40.815	40.94	37.705	39.829
	30	294.629	339.91	397.47	320.735	296.749	442.459

**Table 4:** Computational time of each algorithm—Part II.

Algo.	Dim.	F7	F8	F9	F10	F11	F12
**OGL-WPA**	2	2.148	1.689	1.042	2.667	1.245	3.241
	5	4.913	3.52	2.558	9.843	4.544	11.542
	10	9.577	7.297	6.31	26.326	12.883	33.248
	30	38.945	31.309	33.09	173.54	72.544	157.541
**ACO**	2	0.29	0.435	0.466	1.071	0.978	1.921
	5	0.762	1.046	0.954	3.532	2.722	4.581
	10	1.345	1.83	1.589	9.227	7.821	12.441
	30	4.721	6.104	5.674	74.516	45.548	73.954
**PSO**	2	0.258	0.252	0.23	0.648	0.712	0.821
	5	0.487	0.526	0.464	2.012	2.124	2.459
	10	0.791	1.127	0.976	6.464	5.887	7.241
	30	2.481	3.484	3.059	52.312	40.814	55.631
**GWO**	2	0.214	0.289	0.229	0.67	0.692	0.721
	5	0.589	0.771	0.64	2.259	2.315	2.812
	10	1.3	1.645	1.379	6.668	6.887	7.354
	30	5.224	6.05	5.605	50.534	51.527	58.972
**BETAGWO**	2	0.997	0.995	0.954	1.397	1.548	1.821
	5	4.118	4.115	3.976	5.468	5.955	7.0581
	10	14.669	14.315	14.208	19.591	21.763	24.542
	30	139.55	123.03	106.73	154.03	167.821	182.972
**WOA**	2	1.382	1.506	1.326	1.745	2.125	2.265
	5	5.655	5.494	4.766	8.533	8.359	8.853
	10	18.894	20.534	18.855	26.028	29.083	29.670
	30	195.772	193.093	113.920	240.921	214.840	210.727
**LSHADE**	2	2.767	2.415	3.083	3.635	4.476	5.527
	5	12.69	12.214	11.58	16.552	14.888	25.139
	10	38.338	39.793	41.004	60.072	54.519	64.823
	30	339.547	341.349	350.002	425.281	479.914	527.511

**Table 5:** Analysis of Wilcoxon statistical test results.

Algorithm	R+	R-	P-value
OGL-WPA vs ACO	335	56	0.0012
OGL-WPA vs PSO	204	75	0.0505
OGL-WPA vs GWO	178	98.5	0.2022
OGL-WPA vs BETAGWO	133	125	0.8596
OGL-WPA vs WOA	144	115	0.4391
OGL-WPA vs LSHADE	107	105	0.9773

## 6 Conclusion

In this paper, we have proposed an improved WPA approach called OGL-WPA. Specifically, we focus on leveraging the existing intelligent techniques, i.e., Opposition-based learning, Genetic algorithm and Levy’s flight, to handle the issues in WPA (e.g., popular initialization, local optimum and convergence speed). To the best of our knowledge, this is the first work on how to improve the WPA in a comprehensive way. We have given the detailed design and implementation of OGL-WPA. Moreover, we also have compared our approach with some other swarm intelligent algorithms over different test functions with extensive experiments. Our experimental results have shown that the proposed OGL-WPA has better global search and local search capability, especially in the cases for multi-peak and high-dimensional functions.

## Supporting information

S1 Appendix Additional experimental results. The results about the comparison of different dimensions are available at 10.5281/zenodo.5109519.
(PDF)
