# Supplementary material for: An improved Wolf pack algorithm for optimization problems: Design and evaluation
Source: PLoS One. 2021 Aug 26;16(8):e0254239. doi: 10.1371/journal.pone.0254239 (PMC8389437; doi:10.1371/journal.pone.0254239)
Supplement: S1 Appendix — The results about the comparison of different dimensions are available at 10.5281/zenodo.5109519. (PDF) [file pone.0254239.s001.pdf]

An Improved Wolf Pack Algorithm for Optimization Problems: Design and Evaluation (Appendix)

Xuan Chen[1], Feng Cheng[2*], Cong Liu[3], Long Cheng[4], Yin Mao[5]

1 Design and Art Branch, Zhejiang Industry Polytechnic College, Zhejiang, China
2 School of Mathematics, Southwest Jiaotong University, China.
3 School of Computer Science, Shandong University of Technology, China.
4 North China Electric Power University in Beijing, and Insight SFI Research Centre for Data Analytics in Dublin.
5 Department of Computer Science, Fordham University in the New York City

* Corresponding author. Email: chengfeng2013@swjtu.edu.cn

0.1 Experimental results

Tables 1-10 show the comparisons of the four indicators of the five algorithms in the 2, 5, 10, and 30 dimensions of the 10 test functions. From the minimum and maximum comparison index, it is found that the value of OGL-WPA algorithm is lower in F1, F2, F3, F4, F6, F8, F9 test function with dimension 2 than that of comparison algorithm, which is only higher in F5, F7 and F10 test function than in β-GWO algorithm. The value of the F1-F10 test function of in dimension 5, 10,30 is lower than that of the contrast algorithm, which indicates the solution quality of OGL-WPA algorithm is good in most cases. According to the Ave. Value, the value of the F1-F10 function is lower than that of the contrast algorithm when the dimension is 2, 5, 30, so the value is higher than that of the PSO algorithm only when the dimension is 10 but is lower than that of other test functions. The OGL-WPA algorithm has good precision. From the comparison of standard deviation, the value of the F1-F10 function of OGL-WPA algorithm is lower than that of other comparison algorithms under the condition of dimension 2, 5, 10 and 30 which explains the better convergence speed of the OGL-WPA .From the comparison results above, this algorithm improves the diversity of population after population initialization, and improves the convergence of the algorithm by selecting the first wolf effectively and adopting Levy behavior, which makes the algorithm perform well in 4 dimensions.

Table 11 shows comparisons of the elapsed time among the five algorithms in test functions. It can be found that OGL-WPA has an increased running time in comparison with other algorithms. The main reason is that OGL-WPA consumes time in population initialization and wolf selection. Although using Levy's flight decreases the local search time, the whole uptime of algorithm becomes longer.

Table 1. Comparison of Different Dimensions in the F1 Function

Algorithm	Dimension n	Min Value	Max Value	Ave. Value	Variance
OGL-WPA	2	1.8005e-09	2.1219e-06	2.1489e-07	3.2456e-07
	5	1.8733e-06	4.9667e-05	1.2589e-05	9.0836e-06
	10	4.8515e-05	0.0007516	0.000228	0.00015
	30	0.00248	0.0182196	0.005999	0.00238
ACO	2	0.0203476	16456.500	1989.67792	4983.5158
	5	0.3707979	30581.9835	4471.44257	8648.6628
	10	562.4510	57141.6827	16120.5908	17758.7147
	30	26114.2603	132024.456	69169.7457	35162.8114
PSO	2	6.3461e-10	0.0021637	0.000139559	0.0003861
	5	0.005738	4.98512	1.01386505	1.3156749
	10	2.29394	481.84602	145.480588	105.66809
	30	1442.3533	6300.0227	3537.511730	1210.68219
GWO	2	2.740278e-07	0.00022655	2.7396458e-05	3.762036e-05
	5	0.0008189	0.05943359	0.0081767	0.0100839
	10	0.0141605	0.4979525	0.1774109	0.1089414
	30	6.904162	33.153283	19.500706	5.9543077
β-GWO	2	1.31884e-08	0.00022771	3.0823552e-05	3.9395170e-05
	5	0.000479	0.03981812	0.0074079	0.00715516
	10	0.039159	0.49914867	0.1783383	0.11130467
	30	6.905039	33.7413895	19.527661	6.00519511

Table 2. Comparison of Different Dimensions in the F2 Function

Algorithm	Dimension n	Min Value	Max Value	Ave. Value	Variance
OGL-WPA	2	4.6788e-05	0.00102	0.00027	0.000163
	5	0.00091	0.00624	0.00312	0.001064
	10	0.00832	0.02214	0.01437	0.003336
	30	0.08383	0.14861	0.11945	0.015775
ACO	2	0.171701	90.7335	9.18269	24.75729
	5	0.847493	16850.7147	798.050	2890.376
	10	4.727616	333392446.90	10854563	49590932.044
	30	44551072.64	1.66977e+20	3.41849e+18	2.36061e+19
PSO	2	4.61154e-06	0.00995994	0.00100221	0.00200884
	5	0.004236052	1.2703288	0.2266671	0.2595154
	10	1.0877692	8.44706953	4.5504297	1.89244510
	30	15.01764810	66.971699	32.4898099	10.564354
GWO	2	0.0001384	0.0020792	0.0007216	0.0004789
	5	0.0039677	0.0344367	0.0155093	0.0065253
	10	0.0389009	0.2332112	0.1175955	0.0417651
	30	1.5549388	4.3662240	2.7791748	0.6740869
β-GWO	2	0.00012913	0.00208936	0.00072609	0.00047575
	5	0.00422805	0.034429330	0.01552950	0.006742115
	10	0.03888746	0.233210126	0.11356567	0.042478287
	30	1.59233317	4.366104518	2.77830880	0.666883028

Table 3. Comparison of Different Dimensions in the F3 Function

Algorithm	Dimension n	Min Value	Max Value	Ave. Value	Variance
OGL-WPA	2	1.77255e-07	0.000392	7.26540e-05	7.44813e-05
	5	2.64471e-06	0.002139	0.00037	0.00037
	10	8.61429e-06	0.002065	0.00066	0.00060
	30	4.06432e-05	0.008976	0.00177	0.00189
ACO	2	0.025686	270.41155	38.91627	88.25971
	5	0.018994	1017.43652	96.19119	273.9338
	10	0.176349	3131.65679	280.8745	828.66022
	30	0.159286	13060.0157	758.43687	2825.95712
PSO	2	4.0795811e-11	0.0016373	9.93502e-05	0.00028406
	5	9.6079588e-11	0.00144573	0.0001186	0.00025395
	10	1.91657e-09	0.01397033	0.0005589	0.00206236
	30	8.92441e-11	0.039662004	0.0017298	0.0059671
GWO	2	2.88641e-05	0.0040572	0.00135711	0.0010650
	5	1.43835e-05	0.0682715	0.01137611	0.0145121
	10	0.0005063	0.2047103	0.04217550	0.0410000
	30	0.0115524	3.1563488	0.75601802	0.7360468
β-GWO	2	2.8864111e-05	0.00405729	0.00129487	0.0010510
	5	0.0006467	0.05538321	0.01129903	0.01277357
	10	0.0028476	0.20470933	0.04467555	0.04431984
	30	0.0115512	3.15637842	0.69541680	0.73811930

Table 4. Comparison of Different Dimensions in the F4 Function

Algorithm	Dimension n	Min Value	Max Value	Ave. Value	Variance
OGL-WPA	2	0.000102	0.000979	0.0004079	0.00022919
	5	0.001218	0.006814	0.0038199	0.00145466
	10	0.005724	0.025244	0.0129461	0.00352010
	30	0.07380	0.240923	0.1228409	0.03491179
ACO	2	0.15161	98.9014	40.89212	44.35097
	5	3.97965	98.8703	77.03869	28.64099
	10	28.8742	99.63691	90.60360	12.575049
	30	87.06238	99.83433	96.58320	2.8063077
PSO	2	2.3331863e-05	0.1167497	0.0079861	0.01785393
	5	0.0515829	4.4855845	0.8557691	0.88475519
	10	4.1968123	19.512747	10.652086	3.66702843
	30	15.841233	39.906120	26.712640	4.98594719
GWO	2	0.0001587	0.0137215	0.00352821	0.0026886
	5	0.0130225	0.1344075	0.05184759	0.02551751
	10	0.0932209	1.3046918	0.36033598	0.19607640
	30	3.3728250	7.9894145	5.17729170	1.12082451
β-GWO	2	0.00013810	0.0104685	0.003603730	0.00265845
	5	0.01302256	0.1074601	0.051506911	0.02447575
	10	0.09322015	1.3046908	0.363599740	0.19894765
	30	2.77459856	7.9894140	5.162038301	1.19848206

Table 5. Comparison of Different Dimensions in the F5 Function

Algorithm	Dimension n	Min Value	Max Value	Ave. Value	Variance
OGL-WPA	2	2.85552e-07	0.000188	3.81922e-05	4.44986e-05
	5	0.00178	32.66563	2.449445	5.11299
	10	0.02726	11.55529	2.097849	2.25198
	30	1.24227	134.5876	34.45741	34.1515
ACO	2	0.1722043	79962962.92	7491663.86	22733221.52
	5	18.845310	167820742.75	23591724.06	55016275.21
	10	259.87970	323733723.58	45288706.35	99657763.65
	30	50005.033	776897069.35	144735089.29	274523195.49
PSO	2	4.536701e-07	7.70186	0.2493286	1.10263
	5	1.6381553	1731.353	165.48465	360.10007
	10	216.66020	67022.206	8255.2939	13456.5928
	30	135286.400	2905425.250	847467.368	634605.300
GWO	2	2.8248945e-07	0.0001932	4.142770e-05	4.738544e-05
	5	0.0347995	4.6612108	2.252820	1.485809
	10	5.6309060	12.4789171	9.6771086	1.174687
	30	51.902806	170.387384	92.029587	23.22154
β-GWO	2	5.2858008e-08	0.00075780	7.0684870e-05	0.0001225
	5	0.0349170	4.66121380	2.3129912	1.4970110
	10	5.6318434	12.4783746	9.6774197	1.1742258
	30	51.902005	170.388762	92.029611	23.221242

Table 6. Comparison of Different Dimensions in the F6 Function

Algorithm	Dimension n	Min Value	Max Value	Ave. Value	Variance
OGL-WPA	2	4.82180e-10	9.29733e-07	2.09207e-07	2.16043e-07
	5	1.71394e-06	5.87215e-05	1.59973e-05	1.23256e-05
	10	4.14823e-05	0.000549	0.00021	0.00012
	30	0.002438	0.013420	0.00611	0.00219
ACO	2	0.03378920	16638.3892	1698.4606	4631.1454
	5	0.31684722	30570.9268	4916.7122	9140.7732
	10	611.799391	56516.6916	16125.4211	17752.7511
	30	26202.1263	132628.8162	69217.9809	35142.1199
PSO	2	2.242653e-10	0.00459714	0.00033719	0.00085328
	5	0.0004307558	12.333401	1.12429536	2.12086752
	10	12.1117574	560.90399	143.552158	115.685413
	30	1363.98861	7473.0983	3643.11901	1311.62557
GWO	2	5.11948e-08	0.0001156	2.851317e-05	2.73035e-05
	5	0.000511	0.0139887	0.004852	0.003110
	10	0.038322	0.5918692	0.186834	0.119432
	30	11.08346	30.331938	19.17017	4.696073
β-GWO	2	5.188776e-08	0.00011561	2.5702547e-05	2.4974229e-05
	5	0.000340	0.01681782	0.0048192	0.0033328
	10	0.033766	0.59184899	0.1816714	0.1182955
	30	11.08352	30.3320805	19.164739	4.6750831

Table 7. Comparison of Different Dimensions in the F7 Function

Algorithm	Dimension n	Min Value	Max Value	Ave. Value	Variance
OGL-WPA	2	5.47779e-08	1.24096e-05	2.97501e-06	3.34417e-06
	5	2.55035e-05	0.00070	0.00021	0.00015
	10	0.00222	5.97331	1.72416	1.16982
	30	2.04969	26.7482	11.2121	5.12330
ACO	2	1.7772	55.2792	25.3269	13.1594
	5	33.0470	133.3064	81.6386	18.5751
	10	88.0778	239.8591	173.6402	35.0953
	30	484.4055	645.3189	544.7705	37.1596
PSO	2	7.322583e-09	1.0012715	0.1596454	0.350144
	5	2.005007	22.881284	8.2732804	4.629569
	10	14.86362	62.029896	34.954967	11.06757
	30	120.8475	264.78830	201.04720	28.58588
GWO	2	1.44108e-07	0.0001148	1.834114e-05	2.29459e-05
	5	0.000635	7.3094649	2.987942	1.961736
	10	2.043079	48.276514	17.40869	12.63959
	30	31.07874	233.17123	118.3956	69.370107
β-GWO	2	1.182404e-08	0.0001142	1.7299998e-05	2.4427828e-05
	5	0.001142	7.3094699	2.922149	1.9029195
	10	2.043013	48.273818	16.96334	12.506471
	30	29.06587	233.17038	114.7511	71.231511

Table 8. Comparison of Different Dimensions in the F8 Function

Algorithm	Dimension n	Min Value	Max Value	Ave. Value	Variance
OGL-WPA	2	0.00027	0.003488	0.00135	0.00077
	5	0.00292	0.014121	0.00789	0.00293
	10	0.00814	0.026876	0.01713	0.00427
	30	0.03324	0.062769	0.04827	0.00678
ACO	2	1.6889	21.76511	17.16632	6.64093
	5	18.9447	21.69285	20.64366	0.65206
	10	19.4543	21.61511	20.84760	0.39061
	30	20.7290	21.42201	21.04721	0.16063
PSO	2	8.395351e-05	0.1240424	0.0098718	0.0202415
	5	0.0183400	4.1681288	1.9215949	1.1884333
	10	3.0564615	10.039153	6.4053804	1.6950190
	30	9.4797347	15.245363	12.033790	1.2499723
GWO	2	0.000172	0.011201	0.0039112	0.002829
	5	0.011834	0.152648	0.0479688	0.027839
	10	0.118492	1.2374913	0.3267526	0.21861631
	30	1.754597	3.8467824	2.9454203	0.40191428
β-GWO	2	0.0002898	0.0134289	0.0045463	0.00335943
	5	0.0118460	0.1526140	0.0461077	0.02794481
	10	0.1184841	1.5621638	0.3642791	0.28184649
	30	1.7545989	3.8467740	2.9298113	0.41075436

Table 9. Comparison of Different Dimensions in the F9 Function

Algorithm	Dimension n	Min Value	Max Value	Ave. Value	Variance
OGL-WPA	2	1.68806e-07	1.92279e-06	5.93896e-07	3.30489e-07
	5	3.54352e-06	0.01175	0.00409	0.00402
	10	5.07908e-05	0.01540	0.00837	0.00405
	30	0.00020	0.04130	0.00874	0.00886
ACO	2	0.022491	1.994289	0.390329	0.471301
	5	0.089352	1.054771	0.411349	0.284688
	10	0.929220	1.131939	1.084629	0.032856
	30	1.132125	1.1816641	1.159412	0.010966
PSO	2	0.0001525	0.0381121	0.017771	0.009673
	5	0.0438146	0.3836333	0.150870	0.0723825
	10	0.1934204	0.8805399	0.528817	0.1916648
	30	1.0162371	1.0399052	1.029196	0.0054230
GWO	2	4.58977e-07	0.008887	0.001384	0.002382
	5	0.05613	0.19672	0.12426	0.03146
	10	0.09968	0.73528	0.54092	0.10674
	30	0.23725	0.98854	0.41591	0.15055
β-GWO	2	2.278180e-07	0.008887	0.001296	0.00229
	5	0.0561339	0.196720	0.125130	0.03284
	10	0.0996871	0.735281	0.5373313	0.12433
	30	0.2372754	0.971461	0.4220914	0.14777

Table 10. Comparison of Different Dimensions in the F10 Function

Algorithm	Dimension n	Min Value	Max Value	Ave. Value	Variance
OGL-WPA	2	1.47145e-06	0.000572	9.33785e-05	0.000122
	5	1.22936e-11	5.495312e-05	7.09154e-06	1.148543e-05
	10	3.81079e-10	1.395420e-05	2.01339e-06	3.429176e-06
	30	1.01920e-11	6.410171e-07	1.41122e-07	1.532696e-07
ACO	2	0.35206	303120900.74	15094438.87	61049304.295
	5	0.23918	530948881.39	36257185.61	125880546.27
	10	0.04124	964022573.85	102621178.35	261018818.43
	30	92918.31	2179533421.39	304714873.44	689903987.10
PSO	2	1.68014e-09	0.2588090	0.0139264	0.03969680
	5	1.82104e-07	0.0286885	0.0020899	0.00574737
	10	8.31437e-10	53.872189	1.6078264	8.39749468
	30	4.24035e-07	260726.74	18682.353	42142.8437
GWO	2	6.33155e-05	0.012798	0.002738	0.002435
	5	3.30133e-05	0.004957	0.001030	0.001033
	10	7.87573e-06	0.001855	0.0004393	0.000479
	30	2.758760e-06	0.00046	8.662211e-05	9.191790e-05
β-GWO	2	7.152411e-05	0.007623	0.0022814	0.0018622
	5	2.533594e-05	0.004958	0.0011217	0.0011092
	10	1.896208e-06	0.0018549	0.0004660	0.0004763
	30	2.142452e-07	0.0004634	8.4814786e-05	8.9742677e-05

Table 11. Time Comparison of 5 Algorithms in Test Functions

Algorithm	Dimension n	$F1$	$F2$	$F3$	$F4$	$F5$	$F6$	$F7$
OGL-WPA	2	1.424	2.036	2.390	1.287	1.507	1.2040	2.148
	5	2.647	4.038	8.249	2.742	6.084	3.1370	4.913
	10	4.863	7.810	26.695	5.396	13.011	4.960	9.577
	30	15.95	27.58	236.85	18.159	75.258	17.927	38.94
ACO	2	0.289	0.3670	0.6110	0.2840	0.3250	0.2280	0.290
	5	0.646	0.7170	1.3820	0.6290	1.1190	0.5950	0.762
	10	1.239	1.3870	3.8580	1.2700	1.9210	1.6720	1.345
	30	3.361	3.7150	22.700	3.9940	8.2000	3.7020	4.721
PSO	2	0.192	0.1720	0.2790	0.1610	0.2100	0.1480	0.258
	5	0.359	0.3460	0.8070	0.3380	0.5270	0.3970	0.487
	10	0.995	0.9900	2.4840	0.6830	1.1760	0.7320	0.791
	30	2.247	2.7110	16.536	1.9310	5.1980	1.9230	2.481
GWO	2	0.255	0.206	0.302	0.191	0.340	0.223	0.214
	5	0.521	0.5450	1.005	0.536	0.694	0.531	0.589
	10	1.098	1.239	2.908	1.198	1.619	1.174	1.300
	30	4.475	4.778	19.29	4.782	7.936	4.793	5.224
β-GWO	2	0.734	1.083	1.156	0.783	0.808	0.924	0.997
	5	4.153	4.139	5.847	4.004	4.605	4.198	4.118
	10	12.582	12.176	14.755	13.709	15.659	14.997	14.66
	30	103.31	104.15	126.333	102.097	113.266	145.464	139.5
